# The usefulness of cardiac CT in the diagnosis of perivalvular complications in patients with infective endocarditis

**DOI:** 10.1007/s00330-018-5965-2

**Published:** 2019-01-14

**Authors:** Tomasz Hryniewiecki, Karina Zatorska, Elżbieta Abramczuk, Dariusz Zakrzewski, Piotr Szymański, Mariusz Kuśmierczyk, Ilona Michałowska

**Affiliations:** 1 Department of Acquired Cardiac Defects, Institute of Cardiology, 42 Alpejska St., 04-081 Warsaw, Poland; 2 Department of Cardiac Surgery and Transplantology, Institute of Cardiology, Warsaw, Poland; 3 Department of Radiology, Institute of Cardiology, Warsaw, Poland

**Keywords:** Endocarditis, Echocardiography, Echocardiography, transesophageal, Tomography, X-ray computed, Prostheses and implants

## Abstract

**Objectives:**

The aim of the study was to compare the usefulness of cardiac CT to transthoracic (TTE) and transesophageal (TEE) echocardiography in the diagnosis of infective endocarditis (IE) and perivalvular complications using surgical inspection as the gold standard.

**Material and methods:**

Fifty-three consecutive patients (42 men, mean age 58.3 ± 12.5) with IE requiring surgical procedures were enrolled in the study. All patients underwent preoperative TTE, TEE, and CT. The presence of vegetations, perivalvular abscess/pseudoaneurysm, leaflet perforation, inflammatory infiltration, and prosthesis dehiscence was assessed.

**Results:**

We analyzed 71 affected valves (58 native, 13 prosthetic). Intraoperative assessment revealed 11 abscesses/pseudoaneurysms. Sensitivity and specificity of echocardiography (TTE + TEE) and CT were 63%, 90% and 81%, 90%, respectively. The combination of CT and echocardiography allowed diagnosing all abscesses/pseudoaneurysms. Inflammatory infiltration was found intraoperatively in 15 patients. Sensitivity and specificity of TEE and CT were 53%, 94% and 46%, 100%, respectively. Intraoperative assessment revealed leaflet perforation in 16 patients. Sensitivity and specificity of TEE and CT were 75%, 79% and 43%, 89%. The sensitivity of the combination of TTE + TEE + CT was 81%. Perivalvular leakage was found in eight patients with a prosthetic valve. Sensitivity and specificity of echocardiography and CT were 100%, 100% and 88%, 100%, respectively. TEE showed higher sensitivity (97%) than CT (89%) in the diagnosis of vegetations.

**Conclusions:**

The combination of TTE, TEE, and CT increased the sensitivity for the detection of valvular and perivalvular complications of IE.

**Key Points:**

*• CT is a useful modality in the diagnosis of IE and its local complications in addition to echocardiography.*

*• For the detection of abscesses and pseudoaneurysms, CT is superior to echocardiography. Combining these two modalities can increase the sensitivity of diagnosing abscess/pseudoaneurysm up to 100%.*

*• Adding CT to TEE increases the sensitivity for detection of inflammatory infiltrate. CT is not superior to echocardiography in diagnosing vegetations, valvular leaflet perforations, and perivalvular leaks, but it can be a useful tool when echocardiography is indeterminate.*

## Introduction

Infective endocarditis (IE) is an infection of the endocardium involving the valves and adjacent cardiac structures, caused by a wide variety of bacteria and fungi [1]. Echocardiography is still the gold standard in IE diagnosis. Although transesophageal echocardiography (TEE) sensitivity is high in diagnosing vegetations and perivalvular complications, negative findings do not rule out IE. An approximative false negative rate of 15% is observed, in part related to the acoustic shadow of artificial valves [2, 3]. Besides, TEE is an invasive examination, which can be associated with complications resulting from the insertion of the probe.

The 2015 guidelines of the European Cardiac Society on IE management included perivalvular lesions visualized in cardiac CT as the main diagnostic criteria [4]. However, these recommendations are based on few studies performed on small groups of patients [5–7]. Other imaging methods are MRI, PET-CT, and leucocytes labeled SPECT-CT [8–10]. Both PET-CT and SPECT-CT were also included in the major criteria for the diagnosis of IE in the latest guidelines. The aim of the present study was to assess the usefulness of cardiac CT in IE patients and to compare it with echocardiography and intraoperative findings for the diagnosis of perivalvular complications.

## Methods

The study was approved by the Bioethics Committee at the Institute of Cardiology. It was conducted in accordance with the principles in the Declaration of Helsinki of 1964.

The study included 71 patients with IE diagnosed based on modified Duke criteria who were hospitalized at the Institute of Cardiology between 2011 and 2015. A CT examination was performed in all patients who had indications for surgical treatment and in those whose echocardiography was unclear. Each patient underwent transthoracic echocardiography (TTE), TEE, and cardiac CT. We analyzed 53/71 patients who underwent a surgery. The mean time and the standard deviation between examinations were 4.3 ± 5.1 days between TTE and CT, 3.9 ± 4.7 days between TEE and CT, and 8.3 ± 12.1 days between CT and surgery.

### Echocardiography

Both TTE and TEE were performed with Philips HD 15 and General Electric Vivid 9 devices. Echocardiographic examinations were performed with state-of-the-art probes and according to the clinical guidelines. Echocardiograms, including Doppler measurements, were done according to current recommendations [11–15]. The diagnosis of complications of IE in echocardiography was in accordance with the definitions contained in the 2009 ESC Guidelines (see Table 1) [16]. A physician with 25 years of experience in echocardiography interpreted the results.

**Table 1:** Definitions of perivalvular complications

	Echocardiography [16]	Computed tomography [1]
Vegetations	Oscillating or non-oscillating intracardiac masses on the valve or other endocardial structures, or on implanted intracardiac material.	Regular, soft tissue masses attached to the valves or other endocardial structures observed in more than two different dimensional views.
Perivalvular abscess	Thickened, non-homogeneous perivalvular area with echodense or echolucent appearance.	Fluid collection surrounded by a thick layer of inflammatory enhancing tissue.
Pseudoaneurysm	Pulsatile perivalvular echo-free space, with color Doppler flow detected.	Space filled with contrast close to the valve communicating with cardiac chambers or the aortic root.
Inflammatory infiltration	Thickened perivalvular area with normal echogenicity	Thick layers of enhancing tissue
Leaflet perforation	Interruption of endocardial tissue continuity traversed by color Doppler flow.	Lack of continuity of the leaflet of the valve. The defect was observed in two different dimensional views.
Prothesis dehiscence	Paravalvular regurgitation identified by TTE/TEE with or without rocking motion of the prosthesis.	Rocking motions of the prosthetic valve of > 15^o^ on cine CT images.

### Computed tomography

ECG-gated CT angiography was performed with a dual source CT Somatom Flash (Siemens Healthineers).

#### Study parameters

First, a non-enhanced prospectively ECG-triggered scan was performed from the carina to the apex of the heart. Secondly, retrospective ECG-triggered CT angiography was performed (beam collimation 2 × 64 × 0.6, 128 slices, lamp rotation time 280 ms, lamp amperage 100–140 kV depending on a patient’s weight, slice thickness 0.6 mm). Unless contraindicated, patients with a heart rate of > 75 beats per minute were administered metoprolol at 2.5–5 mg intravenously. Since they initially qualified for surgery, all patients underwent coronary CT angiography. The patients without contraindications were given nitroglycerin sublingually. CT angiography was performed after intravenous administration of high-iodine concentration contrast medium (≥ 350 mg/ml) into the basilic vein at 5–6 ml/s in the amount of 70–100 ml (depending on a patient’s weight) followed by 30 ml of saline. In order to calculate the time for starting data acquisition, each patient was administered a 10-ml bolus of the contrast medium. When IE of tricuspid or pulmonary valve was suspected, the contrast medium was administered according to a special protocol so that all the heart chambers could be filled with contrast. The field of scanning ranged from the tracheal bifurcation to the base of the heart. In selected cases (when infiltration or abscess formation was suspected by angio-CT scans), delayed acquisition was performed. The analysis included the presence and the size of vegetations and such complications as perivalvular abscesses/pseudoaneurysms, leaflet perforations, and inflammatory infiltration; in patients with prosthetic valve endocarditis (PVE), prosthesis dehiscence was additionally assessed (Table 1).

### CT image analysis

The dataset of contrast-enhanced CT scan was reconstructed every 5% of the R–R interval and analyzed using the dedicated software syngo.via (Siemens Healthineers). Several views of the aortic, mitral, tricuspid, and pulmonary valves were obtained by using the multiphase source data.

The aortic valve closure was assessed at the diastolic phase of the cardiac cycle. The aortic valve was analyzed using three- and four-chamber views and through the aortic valve plane parallel to the transverse plane of the three coronary sinuses.

The morphology of the mitral valve was assessed in the systolic and diastolic phase of the cardiac cycle. The mitral valve was analyzed using four- and two-chamber views, three-chamber views (LVOT), and in-plane views of the mitral annulus.

The tricuspid valve was evaluated on the four- and two-chamber long-axis and RV short-axis views.

The pulmonary valve was analyzed along the pulmonary annulus plane and on RVOT views.

The motion of the vegetation or the rocking motion of the prosthetic valves in patients with paravalvular leakage was shown using cine reconstruction images. A radiologist with wide experience in the diagnosis of infective endocarditis interpreted the results.

### Statistical analysis

The data distribution was verified by the Kolmogorov-Smirnov test. Variables did not have a normal distribution. Consequently, non-parametric tests were used for the statistical analysis. Cohen’s kappa test was used to assess the diagnosis correlation of vegetations and particular perivalvular complications between echocardiography, CT, and intraoperative findings (considered as the gold standard). It was also used to assess intraobserver agreement for echocardiography and CT imaging findings. The Kappa coefficient of 0–0.4 was considered a poor correlation, 0.4–0.75 a good correlation, and 0.75–1.0 a very good correlation of the findings [17]. Sensitivity, specificity, positive predictive value, and negative predictive value of each imaging modality were calculated. Receiver operating characteristic (ROC) analysis was performed to determine sensitivity and specificity of TEE and CT. The correlation of vegetation sizes visualized in TEE vs CT was checked with the Spearman correlation test. *p* < 0.05 was considered statistically significant. The STATISTICA software was used for the analysis.

## Result

### Study group

The study group comprised 53 patients with diagnosed IE of native or prosthetic valves. Table 2 shows the clinical characteristics of patients. The diagnostic value of echocardiography and CT was compared with surgery as the gold standard.

**Table 2:** Patients’ characteristic

Number of patients	53
Age (years)	
• Range	22–84
• Mean/standard deviation	58.3 ± 12.5
Sex	
• Men	42 (79%)
• Women	11 (21%)
Valves affected	71
Aortic valves affected	33/71 (47%)
• Native valve	25/33
• Artificial valve	8/33
Mechanical valve	5/8
Biological valve	3/8
Mitral valves affected	30/71 (42%)
• Native valve	25/30
• Mechanical valve	5/30
Native tricuspid valve affected	6/71 (8%)
Native pulmonary valve affected	2/71 (3%)
Subvalvar localization	3
• Ventricular septal defect	
Etiology	
• Streptococci	15/53 (28%)
• Staphylococci	14/53 (26%)
• Enterococcus faecalis	10/53 (19%)
• Pseudomonas stutzeri	1/53 (2%)
• Corynebacterium	1/53. (2%)
• Cardiobacterium	1/53 (2%)
• Haemophilus influenzae	1/53 (2%)
• Negative blood culture	10/53 (19%)

### Intraobserver readout of echocardiography and CT findings

The intraobserver agreement for the diagnosis of perivalvular complication was *κ* = 1.

### Perivalvular abscesses/pseudoaneurysms

**Fig. 1:**
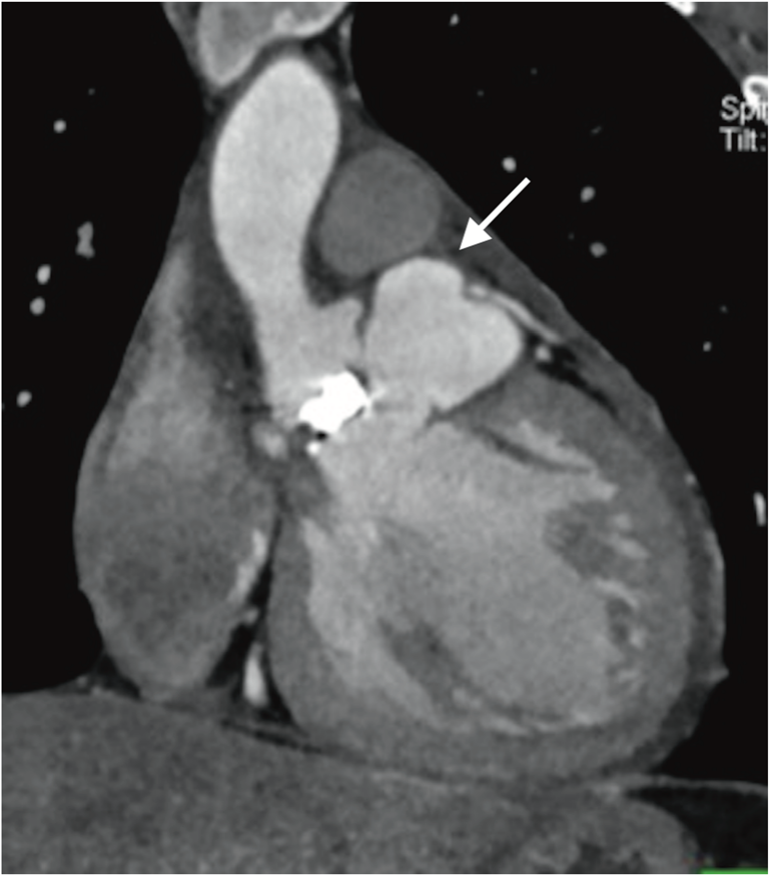
Pseudoaneurysms of the left ventricular outflow tract in patient after aortic valve replacement (arrow). ECG-gated computed tomography, multiplanar reconstruction, frontal oblique view

**Fig. 2:**
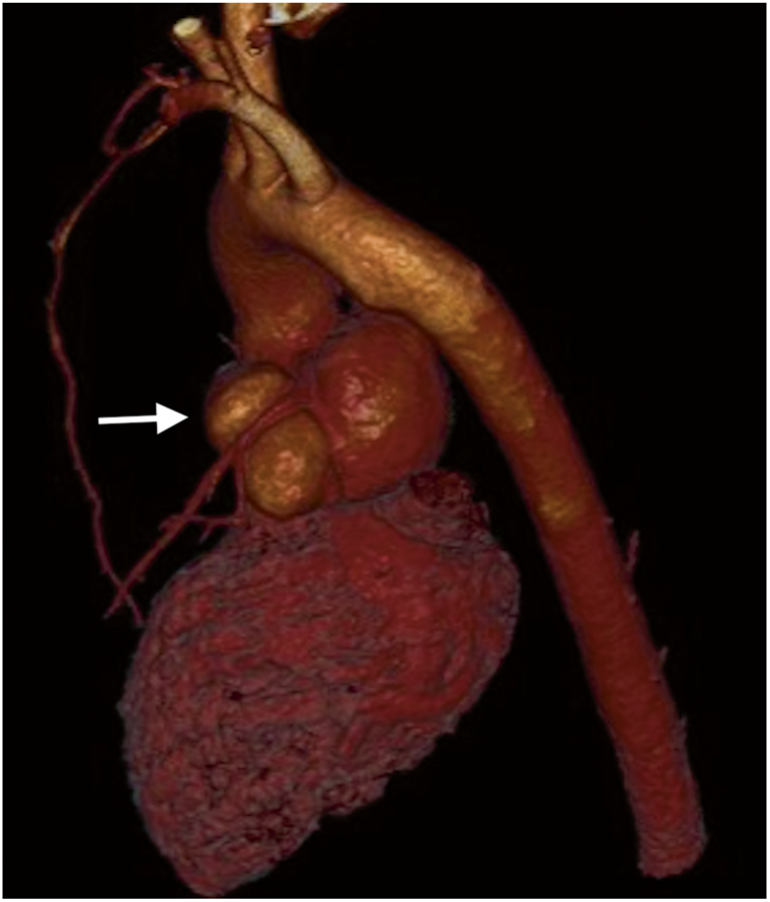
Pseudoaneurysm of the left ventricular outflow tract (arrow). ECG-gated computed tomography, volumetric reconstruction

Eleven perivalvular abscesses/pseudoaneurysms were found intraoperatively—six in patients with prosthetic valve and five in patients with native valve IE. Of these, TTE diagnosed correctly four cases. In two cases, the findings were false positive, and in seven—false negative. In TEE, five patients were diagnosed correctly. The findings were false positive in three cases, in six—they were false negative. When both echocardiography examinations were compared with the findings of intraoperative testing, seven patients were found to have been diagnosed correctly. Sensitivity and specificity of combination of TTE and TEE was 63% and 90%. Adding CT findings to the analysis led to all perivalvular abscesses/pseudoaneurysms being diagnosed correctly (Figs. 1 and 2).

### Inflammatory infiltration

**Fig. 3:**
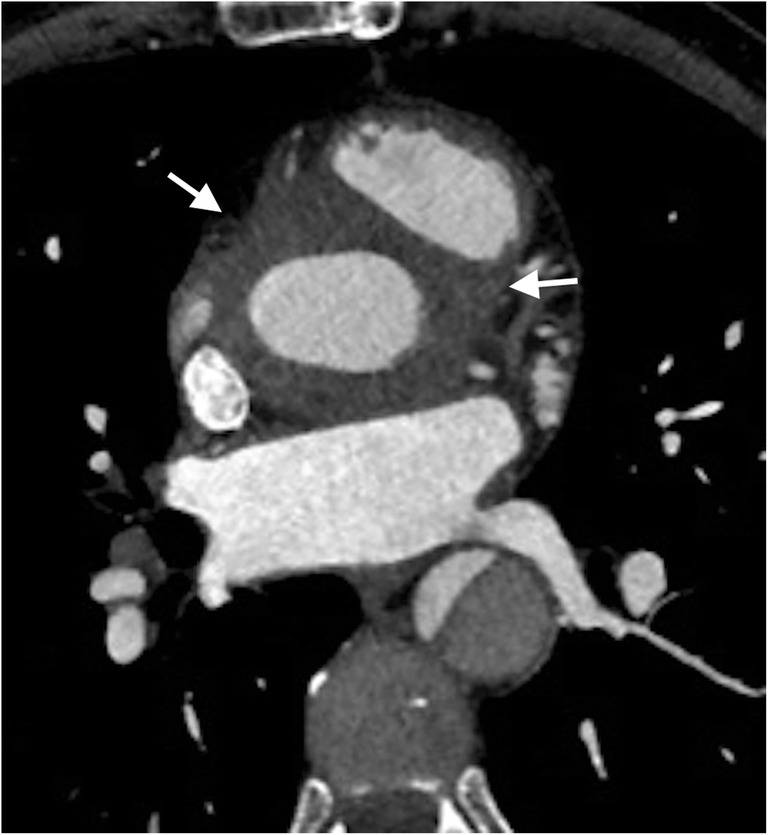
Inflammatory infiltration around the supra coronary graft in patient after type A aortic dissection (arrows). ECG-gated computed tomography, multiplanar reconstruction, axial oblique view

Inflammatory infiltrates were found intraoperatively in 15 cases. In these patients, TTE correctly diagnosed four infiltrations and there were three false positives. The examination failed to visualize the infiltration in 11. Eight patients were diagnosed correctly by TEE and there were false positive in two. No infiltration was found in seven. CT diagnosed correctly seven patients. In eight patients, no infiltration was diagnosed. There were no false positive findings. The highest sensitivity of testing was obtained when combining TEE and CT as 11 patients were diagnosed correctly, while four infiltrations were not detected (Fig. 3).

### Leaflet perforation/destruction

**Fig. 4:**
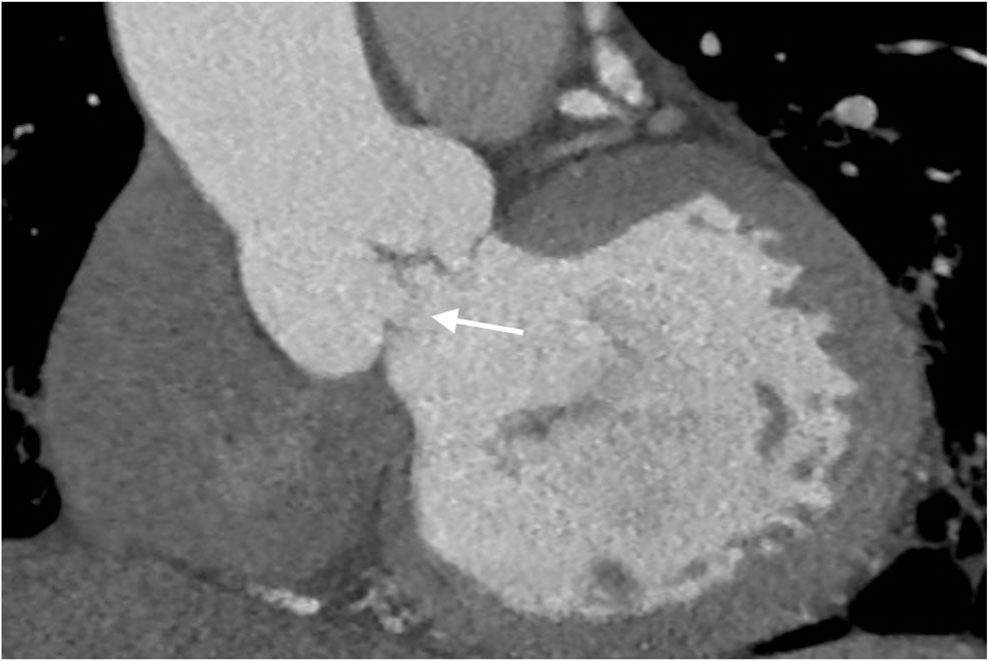
Aortic valve leaflet perforation (arrow). ECG-gated computed tomography, multiplayer reconstruction, frontal oblique view

The analysis covered 45 patients with IE affecting native or biological valves. In 16 of them, perforations were diagnosed intraoperatively. TTE diagnosed perforations correctly in 6/16 and there were five false positives. TEE recognized the lesion correctly in 12 patients, and there were six false positives. Four perforations were not visualized. With CT, seven perforations were visualized correctly and there were three false positives. Combining CT with echocardiography diagnosed perforation in one more patient (Fig. 4).

### Perivalvular leak

The analysis covered 11 patients with mechanical or biological valves. Intraoperatively, a perivalvular leak was found in eight patients. Both TTE and TEE had diagnosed all the leaks that were confirmed intraoperatively. Seven patients were diagnosed by CT. None of the imaging modalities yielded false-positive findings.

### Vegetations

**Fig. 5:**
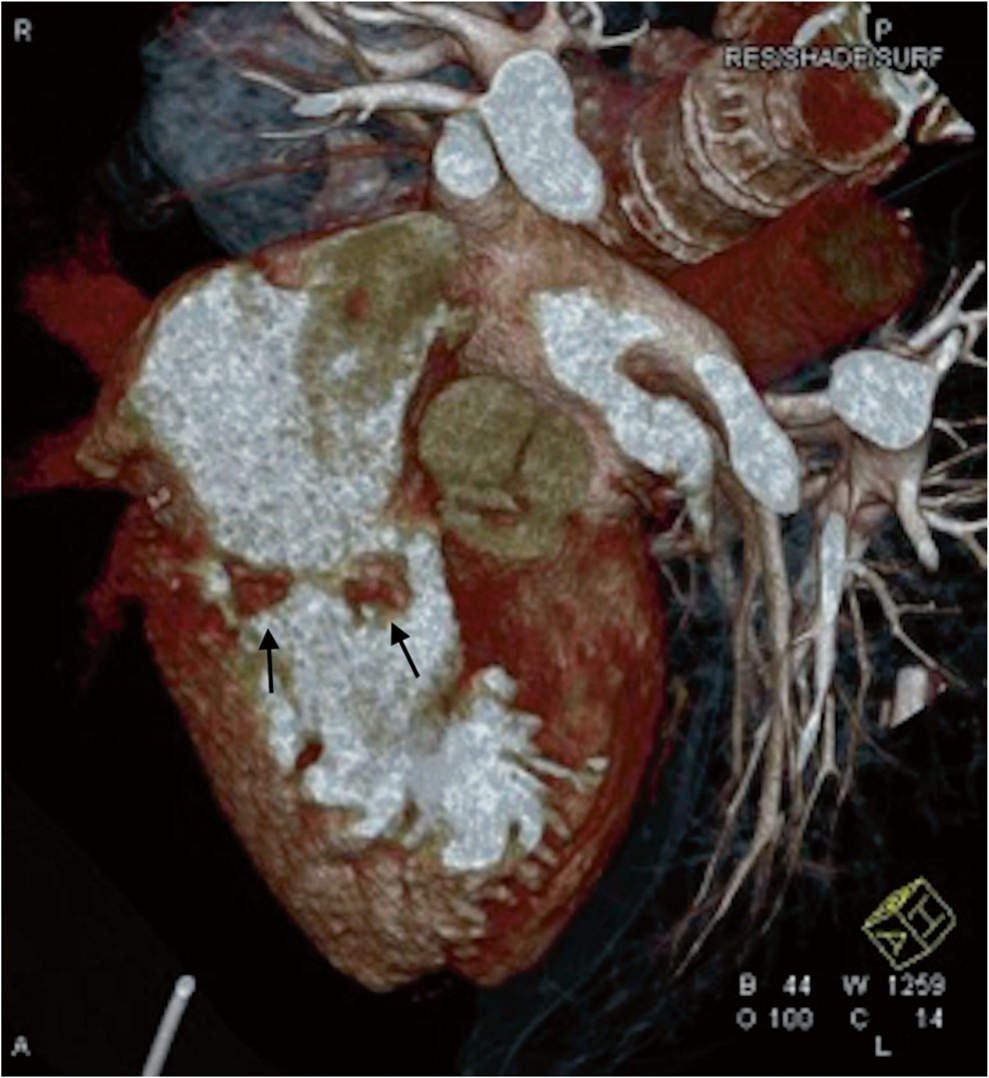
Vegetations on mitral valve (arrows). ECG-gated computed tomography, volumetric reconstruction

Intraoperatively, vegetations were found in 39 patients, 32 of whom were correctly diagnosed with TTE, 38 with TEE, and 35 with CT. There were six false positives with TTE, four false positives with CT, and eight false positive findings with TEE. Combining CT with TEE allowed all the patients with vegetations to be diagnosed correctly (Fig. 5).

### Correlation of vegetation sizes seen in TEE vs CT

The size of vegetations at CT significantly correlated with TEE (*r*_s_ = 0.86; *p* < 0.000001).

**Fig. 6:**
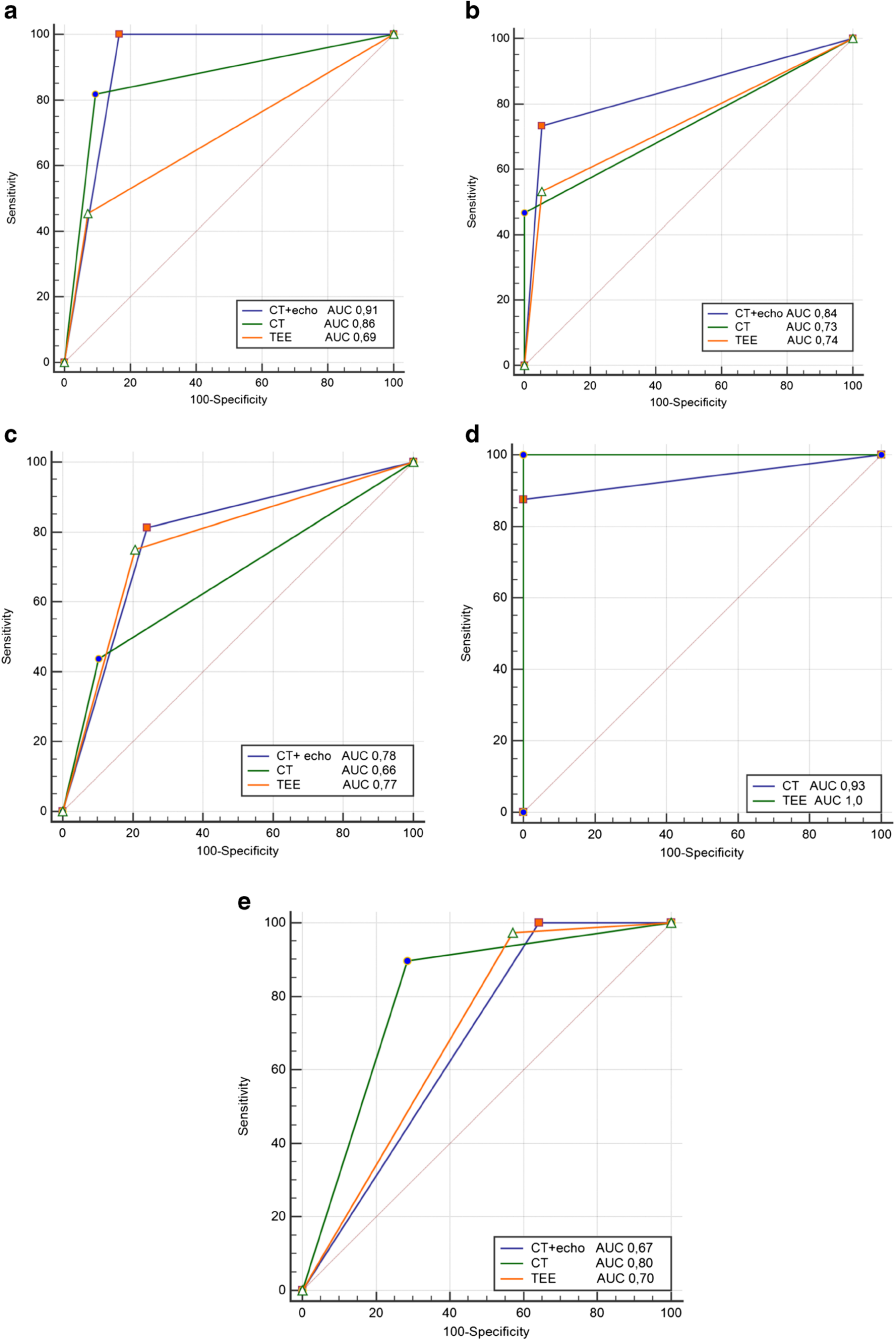
Receiver-operating characteristic curves for transesophageal echocardiogram and electrocardiography-gated contrast- enhanced cardiac computed tomography in the diagnosis of abscess/pseudoaneurysm (**a**), inflammatory infiltration (**b**), perforations (**c**), perivalvular leak (**d**), and vegetations (**e**). CT, computed tomography; TEE, transesophageal echocardiography; AUC, area under curve

The results of the statistical analyses are presented in Table 3 and Fig. 6.

**Table 3:** The results of the statistical analyses

Findings	Sensitivity				Sensitivity specificity				Positive predictive value (PPV)				Negative predictive value (NPV)			
	TTE (%)	TEE (%)	CT (%)	All (%)	TTE (%)	TEE (%)	CT (%)	All (%)	TTE (%)	TEE (%)	CT (%)	All (%)	TTE (%)	TEE (%)	CT (%)	All (%)
Abscess^a^	36	45	81	100	95	92	90	83	66	62	69	61	85	86	95	100
Infiltrate^b^	26	53	46	73	92	94	100	94	57	80	100	84	76	83	82	90
Perforation^c^	37	75	43	81	82	79	89	75	54	66	70	65	70	85	74	88
Leak^d^	100	100	88	–	100	100	100	–	100	100	100	–	100	100	75	–
Vegetation^e^	82	97	89	100	57	42	71	36	84	82	89	81	53	85	71	

^a^TTE (*p* = 0.003; *κ* = 0.38); TEE (*p* = 0.002; *κ* = 0.42); CT (*p* < 0.001; *κ* = 0.67); all (*p* < 0.001; *κ* = 0.67)

^b^TTE (*p* = 0.07; *κ* = 0.22); TEE (*p* < 0.001; *κ* = 0.53); CT (*p* < 0.001; *κ* = 0.55); all = TEE + CT (*p* < 0.001; *κ* = 0.70)

^c^TTE (*p* = 0.13; *κ* = 0.21); TEE (*p* < 0.001; *κ* = 0.52); CT (*p* = 0.01; *κ* = 0.36); all (*p* < 0.001; *κ* = 0.54)

^d^TTE/TEE (*p* < 0.001; *κ* = 1); CT (*p* = 0.007; *κ* = 0.79)

^e^TTE (*p* = 0.005; *κ* = 0.38); TEE (*p* < 0.001; *κ* = 0.47); CT (*p* < 0.001; *κ* = 0.61); all = TEE + CT (*p* < 0.001; *κ* = 0.45)

## Discussion

Perivalvular complications are one of the most severe complications of IE. They worsen the prognosis, the perioperative mortality, the risk for infection recurrence, and the perivalvular leak development [5]. Several studies prove that an early surgery may decrease patients’ mortality rates [5, 18].

In our study, adding TTE/TEE with CT allowed detection of all abscess/pseudoaneurysm. Echocardiography did not visualize this complication in patients with artificial valves or native valve with large calcification. In patients with false positive recognition of abscess/pseudoaneurysm with TEE, an inflammatory infiltrate was found by a surgeon. CT provided additional anatomic information of the extensiveness of abscess which was helpful for planning surgery. CT had also higher sensitivity in diagnosing of inflammatory infiltration. In patients with native or biological valves who underwent surgery, TEE was better than CT in diagnosing of leaflet perforation. But adding CT to echocardiography increased sensitivity of the test to 81%. TEE was superior to CT in detecting perivalvular leak. TEE with color Doppler could detect small paravalvular regurgitation with a slight rocking motion of the prosthesis. CT was less sensitive but had higher specificity in visualizing vegetations than TEE. Size measurements of vegetations with CT showed a good concordance with TEE.

The results of our study are consistent with previous studies. Gahide and Feutchner reported 100% sensitivity of CT in diagnosing abscesses [5, 6]. However, these studies included patients with IE of native valves and few patients with biological valves. Papers on PVE reported 100% sensitivity only for a panel consisting of echocardiography and CT [7, 19, 20]. The recently published papers did not address the question of inflammatory infiltrates in IE patients [5, 6, 19]. Concerning inflammatory infiltrates, Fagman et al [7] analyzed aortic wall thickening on CT and TEE and found a good correlation (*κ* = 0.83). No comparative analysis with the intraoperative findings was performed. The results of the analysis presented the difficulty in diagnosing inflammatory infiltration. Unlike surgery, echocardiography or CT cannot recognize changes in the morphology of inflamed tissue—changes in the color or consistency.

Leaflet perforations are common complications of IE. They are observed in 35–40% of patients with affected left heart valves. Once this condition develops, it is associated with severe valve regurgitation leading to heart failure and is an indication for urgent surgery [21]. CT usefulness in diagnosing perforation was assessed by Feutchner et al [6]. In their study, four patients had perforation diagnosed both by TEE and surgery, none by CT.

The number of valve surgeries is estimated to increase 4–7% yearly; consequently, the problem of PVE increases too [22]. One of the criteria to recognize PVE is the destruction of the valve ring leading to valve dehiscence and perivalvular leak formation. Fagman et al reported that TEE was more sensitive than CT to assess the usefulness of CT in diagnosing perivalvular leaks [7, 20]. There was no correlation with intraoperative examination, though.

One of the main criteria of echocardiographic diagnosis of IE is the presence of vegetations. The accuracy of echocardiography depends on various factors including echocardiography mode, vegetation size, vegetation localization, calcifications, and previous concomitant valvular defects [23]. The causes of false positive findings can be Lambl’s excrescences, a piece of surgical suture found at the ring of an artificial valve, false tendinous cords in the left ventricle, or Chiari’s network in the right atrium [24]. If a vegetation seen in echocardiography is not found during surgery, the reason may be that it tore off the valve, causing an overt or silent peripheral embolism. The usefulness of diagnosing vegetation with CT has been assessed in several studies. Gahide et al reported 71% sensitivity of CT in vegetation diagnosis [5]. When only patients with large (> 10 mm) vegetations were included in the analysis, CT sensitivity was 100%. Feutchner et al reported 96% sensitivity and 97% specificity of CT in comparison with surgery in the “per valve” analysis [6]. Fagman et al reported a medium correlation in vegetation assessment between CT and TEE in a group of patients with IE of prosthetic aortic valves [7]. Habets et al reported sensitivity and specificity of 63% and 100%. Once CT was added, they rose to 100% and 100% [19]. In the 2016 paper by Fagman et al, CT sensitivity was 50% for large vegetations (> 1.5 cm) [20]. Although CT is less sensitive than TEE, it can be helpful in diagnosing vegetations correctly in patients with degenerated or mechanical valves. It also helps to differentiate vegetations from primary heart tumors or thrombi [25]. The possibilities to visualize valvular changes in the right heart are limited because of inhomogeneous enhancement of the right ventricle. In this situation, TTE is the gold diagnostic standard. TEE is done mainly in order to exclude left heart involvement [26].

The present study has some limitations. The main was time between imaging and surgery, which resulted in different duration of antibiotic therapy, potentially influencing correlation. Another limitation is a small group of patients with PVE who underwent surgery. Besides, it was a single-center study.

In conclusion, CT is a useful modality in the diagnosis of IE and its local complications. It detects abscesses and pseudoaneurysms better than echocardiography. Combining the two modalities can increase the sensitivity of diagnosing abscess/pseudoaneurysm up to 100%. Adding CT to TEE increases the sensitivity of diagnosing inflammatory infiltrate. CT is not superior to echocardiography in diagnosing vegetations, valvular leaflet perforations, and perivalvular leaks, but can be a useful tool when echocardiography is non-diagnostic. CT should be considered as a standard in patients with suspected IE. It may provide additional information about perivalvular complications in both prosthetic and native valves.

## Abbreviations

IE: Infective endocarditis
PVE: Prosthetic valve endocarditis
TEE: Transesophageal echocardiography
TTE: Transthoracic echocardiography
